# Metabolism of the Marine Phycotoxin PTX-2 and Its Effects on Hepatic Xenobiotic Metabolism: Activation of Nuclear Receptors and Modulation of the Phase I Cytochrome P450

**DOI:** 10.3390/toxins9070212

**Published:** 2017-07-05

**Authors:** Jimmy Alarcan, Estelle Dubreil, Antoine Huguet, Dominique Hurtaud-Pessel, Stefanie Hessel-Pras, Alfonso Lampen, Valérie Fessard, Ludovic Le Hegarat

**Affiliations:** 1 Toxicology of Contaminants Unit, French Agency for Food, Environmental and Occupational Health & Safety, ANSES, 35306 Fougères, France; jimmy.alarcan@anses.fr (J.A.); antoine.huguet@anses.fr (A.H.); valerie.fessard@anses.fr (V.F.); 2 Analysis of Residues and Contaminants Unit, French Agency for Food, Environmental and Occupational Health & Safety, ANSES, 35306 Fougères, France; estelle.dubreil@anses.fr (E.D.); dominique.pessel@anses.fr (D.H.-P.); 3 Federal Institute for Risk Assessment, Department of Food Safety, Max-Dohrn-Straße 8-10, 10589 Berlin, Germany; Stefanie.Hessel-Pras@bfr.bund.de (S.H.-P.); Alfonso.Lampen@bfr.bund.de (A.L.)

**Keywords:** PTX-2, metabolism, CYP450, nuclear receptors

## Abstract

PTX-2 is a marine biotoxin frequently found in shellfish that can lead to food intoxication in humans. Information regarding PTX-2 metabolism is scarce, and little is known of its effect on xenobiotic-metabolizing enzymes (XME) or its molecular pathways. The aim of this study was consequently to examine PTX-2 Phase I metabolism using rat and human liver S9 fractions, and also to assess the capability of PTX-2: (i) to modulate the gene expression of a panel of Phase I (CYP450) and II (UGT, SULT, NAT, and GST) enzymes, as well as the Phase III or 0 (ABC and SLCO) transporters in the human hepatic HepaRG cell line using qPCR; (ii) to induce specific CYP450 in HepaRG cells measured by immunolabeling detection and the measurement of the cells’ activities; and (iii) to activate nuclear receptors and induce CYP promoter activities in HEK-T and HepG2 transfected cell lines using transactivation and reporter gene assay, respectively. Our results indicate that PTX-2 hydroxylation occurred with both rat and human S9 fractions. Whereas PTX-2 mostly upregulated the gene expression of CYP1A1 and 1A2, no induction of these two CYP activities was observed. Lastly, PTX-2 did not act as an agonist of CAR or PXR. Due to its effects on some key XME, more attention should be paid to possible drug–drug interactions with phycotoxins, especially as shellfish can accumulate several phycotoxins as well as other kinds of contaminants.

## 1. Introduction

**Figure 1:**
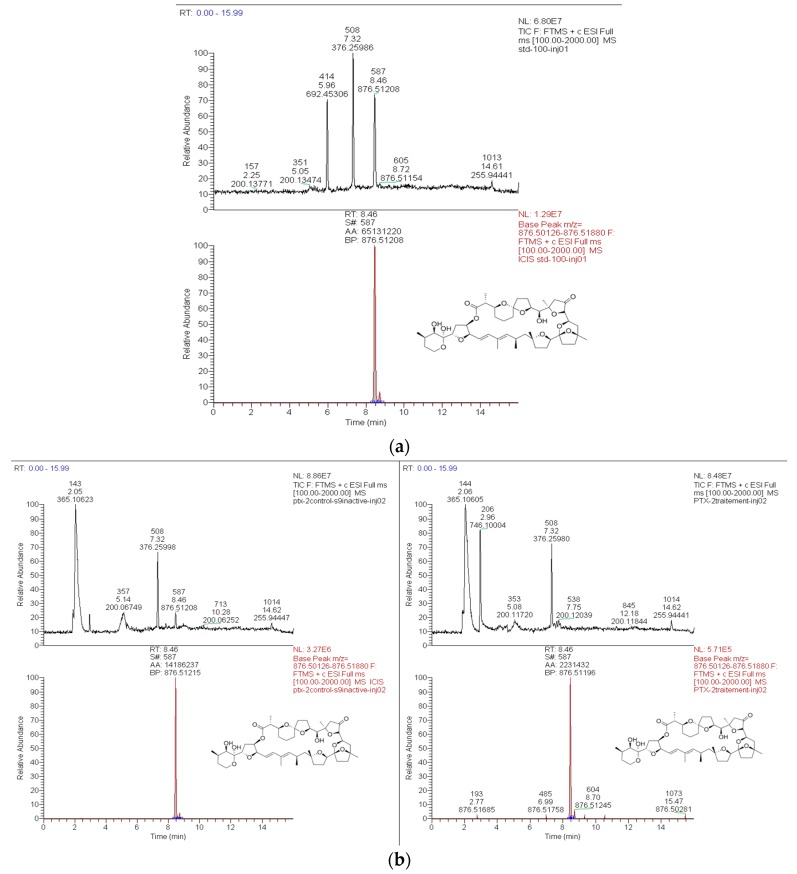
Total Ion Chromatogram (above) and Extracted Ion Chromatogram with extraction window of 5 ppm (below) obtained from the LC–HRMS analysis of PTX-2 in different samples. (**a**) Standard at 100 ng/ml, (**b**) inactivated or active S9.

Pectenotoxins (PTXs) are a group of marine biotoxins whose structure is based on polyether lactones produced by a restricted variety of phytoplanktonic species [1,2,3]. Among this group, pectenotoxin 2 (PTX-2, see Figure 1) is the best documented compound. Although no correlation between PTX-2 contamination and diarrhea has been ascertained [4,5], PTX-2 has been recurrently associated with diarrhetic shellfish poisoning with gastrointestinal symptoms observed in humans. Moreover, the small intestine and the liver have been depicted as the two main target organs of this toxin in rodents, with an increase of intestinal fluid after oral administration to mice [6], and some hepatic damage after intraperitoneal injection [7]. The major deleterious effects of PTX-2 at the cellular level are mediated via actin depolymerization leading to cytoskeleton disruption [8]. The apoptosis or suppression of NF-κB activity has also been described [9,10]. We previously showed that PTX-2 was cytotoxic in the human metabolic competent hepatoma cell line HepaRG, inducing apoptosis and DNA damage [11]. We also showed that PTX-2 failed to induce PXR translocation in HepG2 cells [11]. Regarding liver metabolism, although a decrease of hepatic protein content was observed, no effect on several enzymatic detoxification activities (total CYP450, cytochrome b5, NADPH-cytochrome c reductase, and aminopyrine *N*-demethylase) was detected in mice [12]. In addition, the formation of several hydroxylated metabolites using rat liver S9 supernatants has been described [13]. We also showed that CYP3A4’s inhibition by ketoconazole highly increases the cytotoxicity of PTX-2 in HepaRG cells, suggesting the implication of Phase I metabolism in PTX-2 detoxification [11].

Taken together, these data suggested that PTX-2 could be metabolized by rat S9 fractions and that Phase I metabolism, such as by CYP3A4, participates in reducing the toxicity of PTX-2. However, the role of the human liver’s metabolism and the question as to whether PTX-2 can regulate its own metabolism remains unclear. In fact, the expression of Phase 0, I, II, and III metabolism proteins is orchestrated by several nuclear receptors and transcription factors (AhR, NRF-2, PXR, and CAR) that recognize xenobiotics as ligands [14,15,16,17]. These regulatory processes enable cells to activate detoxification, and protect them from xenobiotics [17].

In this study, we compared the metabolism of PTX-2 by human and rat liver S9 fractions, and we investigated the capability of PTX-2: (i) to modulate the gene expression of a panel of Phase I (CYP450) and II (UGT, SULT, NAT and GST) enzymes, as well as the Phase 0 and III (ABC and SLCO) transporters in the human hepatic HepaRG cell line using qPCR; (ii) to induce specific CYP450 in HepaRG cells measured by immunolabeling detection and the measurement of the cells’ activities; and (iii) to activate nuclear receptors and induce CYP promoter activities in HEK-T and HepG2 transfected cell lines using transactivation and reporter gene assays, respectively.

## 2. Results

### 2.1. PTX-2 Metabolism in Rat and Human S9 Fractions

#### 2.1.1. High Resolution Mass Spectrometry (HRMS) Method for PTX-2 Quantification and the Detection of Metabolites

We first developed a LC–HRMS method for PTX-2 quantification. The chromatographic step was performed as described in the Materials and Methods section. PTX-2 was eluted after the same retention time in both active and inactivated S9 as the standard (Figure 1). The standard solution of PTX-2 was used to establish a linear calibration curve (*R*^2^ = 0.99) between 5 and 100 ng/mL. The limit of detection (LOD) and the limit of quantification (LOQ) were estimated using signal intensities of 5 and 10 ng/mL standards, since no noise level was detected when extracting molecular mass. The LOD was assessed at 0.25 ng/mL and the LOQ at 1.1 ng/mL. Next, we determined the recoveries of PTX-2 following treatment with inactivated S9 fractions. We observed recoveries of 177 ± 2.4% and 151 ± 4.9% with inactivated rat S9, while 116 ± 38.5% and 122 ± 1.9% recoveries were obtained with inactivated human S9. Since the method proved to be efficient for the detection and quantification of PTX-2, we investigated the loss of PTX-2 in active S9 fractions.

#### 2.1.2. Loss of PTX-2 and Metabolite Formation in Active S9 Fractions

We observed more than half of the decrease (53 ± 8.1% and 60 ± 0.3%) of PTX-2 with active rat S9. Regarding active human S9, while 93 ± 1.2% of the PTX-2 disappeared in the first assay, only 50 ± 0.3% of the PTX-2 disappeared in the second assay.

**Figure 2:**
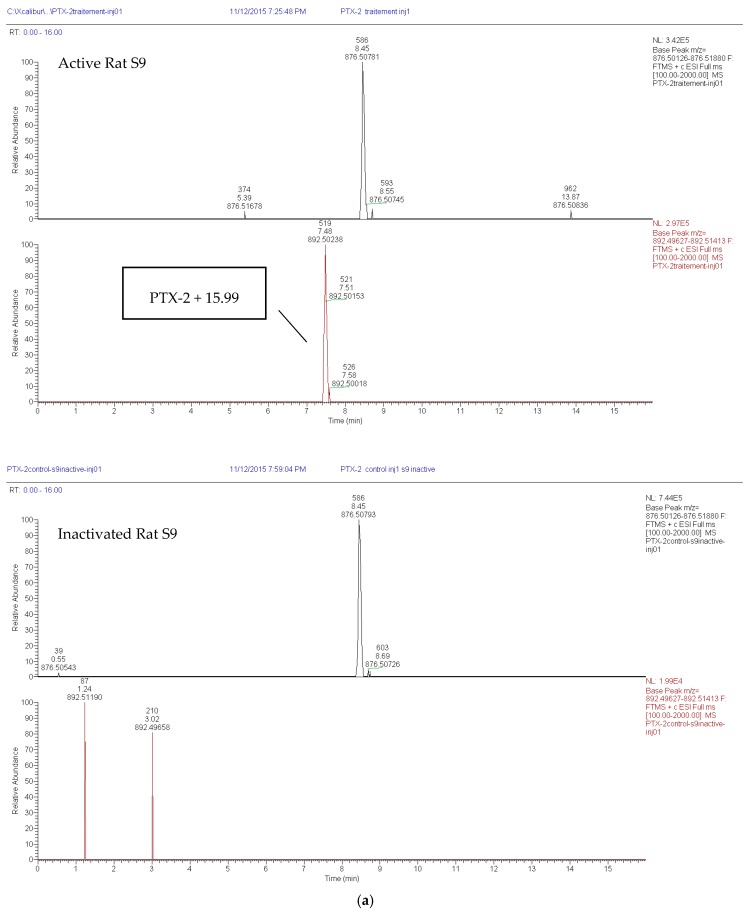

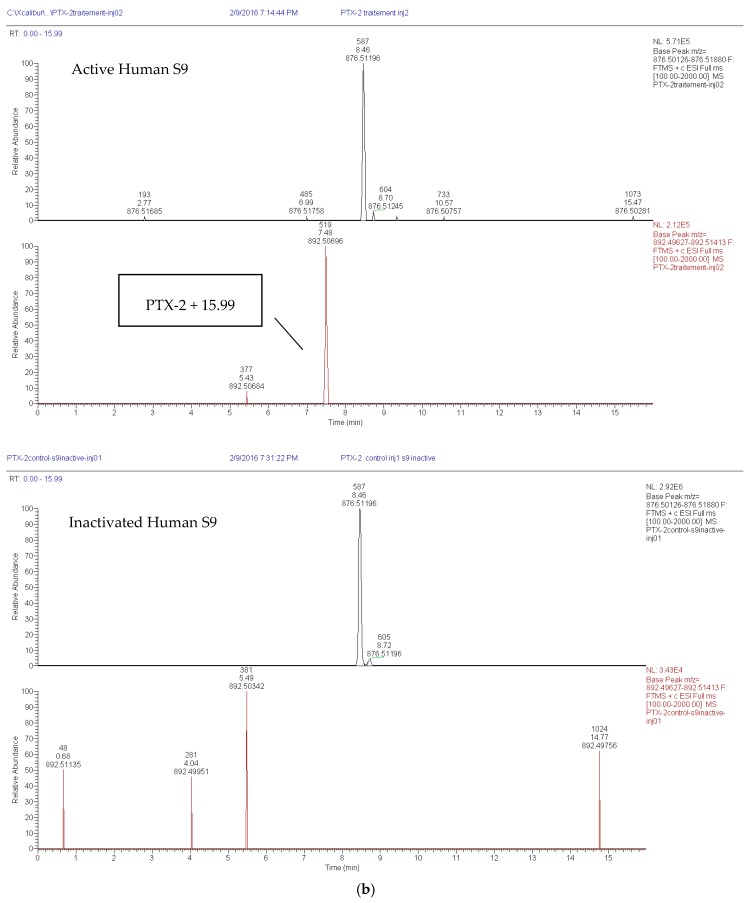
Total ion chromatograms obtained by LC–HRMS analysis. Mass traces of PTX-2 of mass ± 5 ppm and of hydroxylated metabolites are depicted for the treatment with induced rat (**a**) and human (**b**) liver S9.

The formation of metabolites was investigated using MetWorks^®^ 1.3.0. SP1. software (Thermo Fisher Scientific, Waltham, MA, USA). Starting from ammonium as the parent adduct mass (*m*/*z* = 876.51), we screened for a wide panel of Phase I reactions based on mass shifts. For both rat and human S9, at least one hydroxylated metabolite could be found in the active S9 samples. The parent compound was also detected. In the first assay with human S9, two additional metabolites corresponding to double and triple hydroxylation were found, but these two metabolites were not found in the second assay. With both inactivated rat and human S9, only a PTX-2 ammonium adduct could be found (Figure 2a,b).

Since these data strongly suggest a major role for Phase I enzymes in the metabolism of PTX-2, we investigated whether PTX-2 may modulate xenobiotic-metabolizing enzyme (XME) gene expressions in liver cells such as the metabolic competent HepaRG cells.

### 2.2. Effects of PTX-2 on the Expression of Phase 0, I, II, and III Metabolism Genes in HepaRG Cells by qRT-PCR

In order to determine the sub-toxic concentrations of PTX-2 for qRT-PCR analysis, viability was assessed in HepaRG cells by nuclear cell counting using a High Content Analysis. After 24 h of treatment, PTX-2 was toxic only for the two highest doses, resulting in a 27% decrease of cell numbers at 128 nM and 42% at 256 nM (see Figure S1). We selected three sub-toxic concentrations of PTX-2, 16, 32, and 64 nM, for an mRNA expression analysis. PTX-2 had almost no effect regarding the Phase 0 influx transporters SLCO, but induced a concentration-dependent effect on both SLC22A1 and SLC22A3 mRNA (1.8-fold and 2.4-fold, respectively) (Table 1). Regarding CYP450 genes, a concentration-dependent upregulation of CYP1A1, 1A2, 2B6, 2C9, and 2C19 mRNA expression was observed. CYP1A1 and 1A2 mRNA expression were the most induced (18.7-fold and 8.8-fold, respectively). Although some variability was observed between the three independent experiments, induction was obvious when the experimental results were analyzed independently (see Table S1). For Phase II genes, the concentration-dependent induction of SULT1E1 (3.5-fold) and UGT1A1 (2.3-fold) was produced by PTX-2, whereas a concentration-independent induction of UGT1A9 and 2B4 was observed (2.1-fold with 32 nM PTX-2). A slight downregulation was observed for GSTM1 (0.8-fold with 16 and 64 nM PTX-2). Finally, PTX-2 upregulation of all of the Phase III transporters was concentration-dependent, with ABCB1 being the most upregulated (2.1-fold). Omeprazole (50 μM) and rifampicin (10 μM) were used as positive controls for CYP450 gene upregulation. Rifampicin, a well-known CYP3A4 inducer [18,19], upregulated CYP3A4 substantially (29.7-fold), and CYP2B6 and 2C9 slightly (3.2-fold and 2.1-fold, respectively). Omeprazole, a well-known inducer of the CYP1A family [18,19,20], greatly upregulated CYP1A1 and 1A2 (over 100-fold), but also CYP3A4 (12.9-fold), and to a lesser extent CYP2B6 (4.7-fold). Considering the potent upregulation of CYP1A1 and 1A2 gene expression by PTX-2, we investigated whether the effect could be detected in CYP1A’s proteins and enzymatic activities. 

### 2.3. Induction of CYP1A2 Proteins in HepaRG Cells

**Figure 3:**
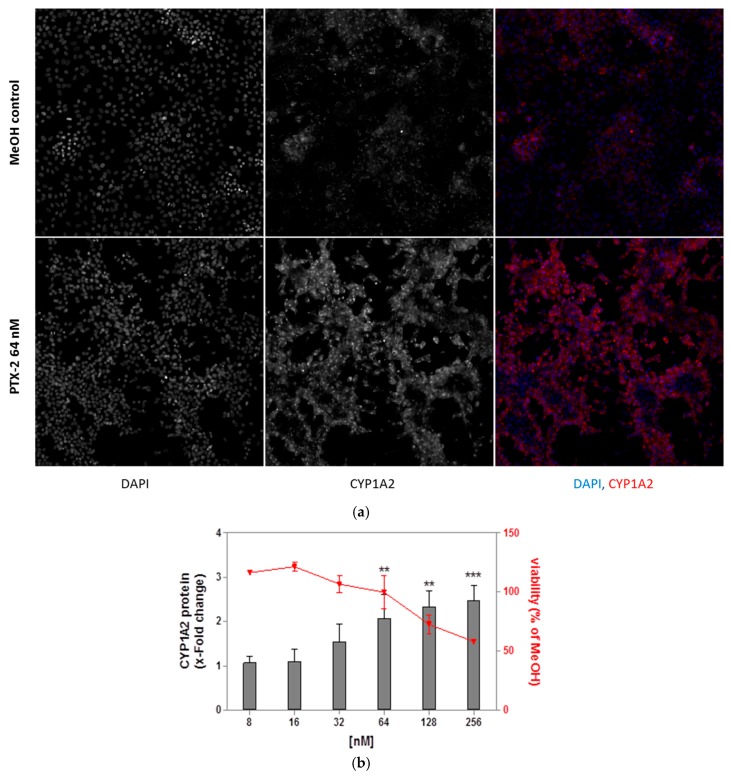
CYP1A2 protein induction after a 24 h treatment with PTX-2 in HepaRG cells. (**a**) Representative images at 10× magnification of CYP1A2 induction in HepaRG control cells (2.57% MeOH) and cells treated with 64 nM PTX-2. CYP1A2 was labeled with a specific antibody, and cell nuclei were stained with DAPI. The images were captured with Arrayscan VTi. Blue: nuclei, Red: CYP1A2; (**b**) CYP1A2 protein induction in HepaRG cells treated with PTX-2 for 24 h. The left Y axis depicts the fold induction of CYP1A2 normalized to solvent control, whereas the right Y axis depicts cell viability. The results were obtained from three independent experiments performed in triplicate (mean ± SD). ** *p* < 0.01, *** *p* < 0.001 after one-way ANOVA followed by Dunnett’s post hoc tests.

In order to confirm the upregulation of gene expression for proteins, CYP1A2 proteins were quantified after a 24 h treatment with PTX-2 by a high content analysis. As shown in Figure 3a, 64 nM PTX-2 greatly induced CYP1A2 fluorescence in comparison with the MeOH solvent control. PTX-2 caused a dose-dependent induction of CYP1A2 (Figure 3b). Although the two highest concentrations induced some toxicity (27% and 42%), a significant induction was also observed for a lower concentration (64 nM), increasing the CYP1A2 protein level 2-fold. Omeprazole poorly induced CYP1A2 proteins (1.3-fold increase).

### 2.4. CYP1A1 Reporter Gene Assay in HepG2 Cells

In order to confirm the upregulation of CYP1A1 mRNA observed in the HepaRG cells, we investigated the capability of PTX-2 to induce CYP1A1 promoter activity in transfected HepG2 cells. Firefly luciferase values were considerably decreased with PTX-2 (in the range of blank values), suggesting some interference of the toxin with the model (data not shown). In order to elucidate this, PTX-2 and a positive control (a mixture of CITCO and 3-MC) were co-incubated. A substantial decrease in luciferase values was again observed, confirming that this model was inappropriate for investigating PTX-2 induction on CYP1A1 due to obvious interference.

### 2.5. Induction of CYP1A Proteins in HepaRG Cells

**Figure 4:**
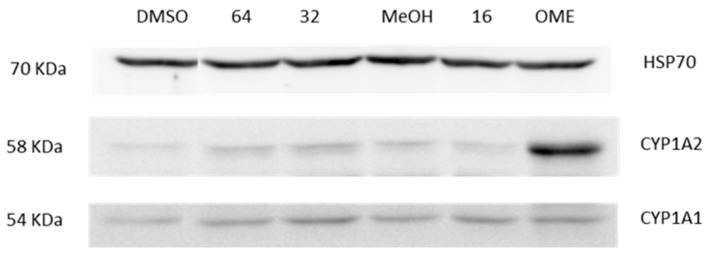
CYP1A induction in HepaRG cells treated with PTX-2. Cells were treated for 24 h with PTX-2 prior to measurement via western blotting (*n* = 1). Omeprazole (50 μM) was used as a positive control.

As we could not investigate CYP1A1 induction via reporter gene assay, we performed western blotting targeting both CYP1A1 and CYP1A2 proteins. Western blotting was performed after a 24 h treatment with PTX-2. As shown in Figure 4, omeprazole greatly induced CYP1A2 proteins but slightly induced CYP1A1. CYP1A2 was slightly induced by 32 and 64 nM PTX-2, while 16 and 32 nM PTX-2 slightly induced CYP1A1.

### 2.6. Effects of PTX-2 on CYP1A Activities in HepaRG Cells

**Figure 5:**
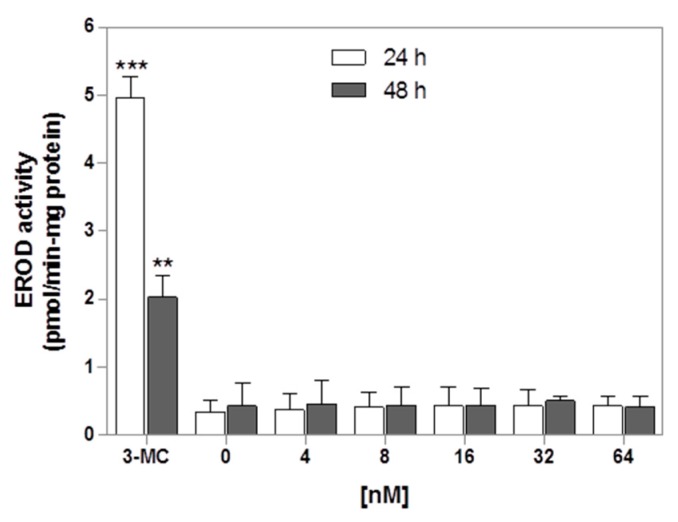
CYP1A activities in HepaRG cells after treatment with PTX-2. Cells were treated for 24 or 48 h with PTX-2 prior to ethoxyresorufin-*O*-deethylase (EROD) activity measurement. The positive control used was 3-methylcholanthrene (5 μM). The results were obtained from two independent experiments performed in triplicate (mean ± SD). ** *p* < 0.01, *** *p* < 0.001 after one-way ANOVA followed by Dunnett’s post hoc tests.

As our results suggest an upregulation of CYP1A1 and 1A2 gene expression, we examined whether PTX-2 could induce these CYP enzymatic activities through a ethoxyresorufin-*O*-deethylase (EROD) reaction. 3-methylcholanthrene (5 μM) was used as a positive control. The results are presented in Figure 5. PTX-2 failed to induce EROD activity regardless of the incubation time in HepaRG cells.

### 2.7. CAR and PXR Transactivation Assay in Transfected HepG2 and HEK-T Cells

**Figure 6:**
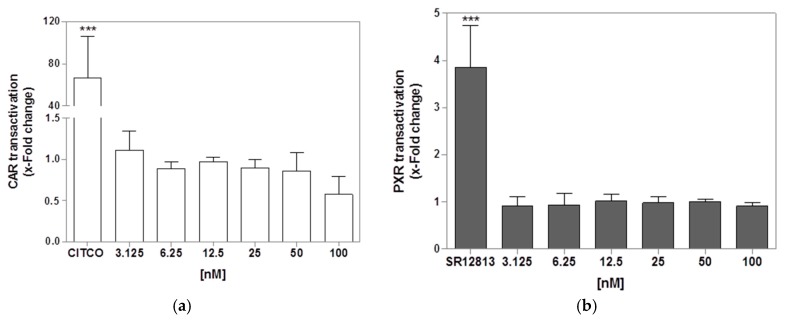
Transactivation of CAR (**a**) and PXR (**b**) in HepG2 and HEK-T cells. The cells were transfected with plasmids before incubation with PTX-2 for 24 h. CITCO (10 μM) and SR12813 (10 μM) were used as positive controls. The results were obtained from three independent experiments performed in triplicate (mean ± SD). *** *p* < 0.001 after one-way ANOVA followed by Dunnett’s post hoc tests.

In order to assess whether PTX-2 could activate nuclear receptors that regulate drug-metabolizing genes, transactivation assays on the two main xenobiotic-metabolizing regulatory nuclear receptors, CAR and PXR, were conducted in transfected HepG2 and HEK-T cells, respectively. PTX-2 was not toxic in either cell line up to 200 nM in the CTB assay. However, PTX-2 induced morphology changes in HepG2 cells (rounded cells, data not shown), so only PTX-2 concentrations below 100 nM were used in the transactivation assays. The results are presented in Figure 6. PTX-2 showed a very slightly inhibited CAR transactivation, and no effects regarding PXR.

### 2.8. AhR Reporter Gene Assay in HepG2 Cells

In order to investigate the capability of PTX-2 to activate AhR, promoter activity was investigated in transfected HepG2 cells. A large decrease of luciferase values was again observed with PTX-2 as well as with a co-incubation of PTX-2 and the positive control 3-MC (data not shown), again indicating the clear interference of PTX-2 with the model.

## 3. Discussion

In this study, we showed that PTX-2 is metabolized by rat and human liver S9 fractions, as the PTX-2 amounts decreased simultaneously with the appearance of at least one hydroxylated metabolite. This metabolite was previously described using a rat S9 fraction [13]. However, we did not detect the four other hydroxylated metabolites that were also reported in this publication. We observed two additional metabolites with human S9, but only in the assay where we observed an almost total loss of PTX-2. We cannot exclude that other metabolites may have been produced that we failed to detect, whether because they were too low in quantity, or not stable enough. In fact, Kittler et al. [13] used a different analytical approach (triple quadrupole mass spectrometry), which could explain the difference with our results. We established recoveries of higher than 100%, which could be explained by ion enhancement phenomena during ionization. Matrix effects have been previously observed when analyzing PTX-2 in mussel extracts [21,22]. Regarding the results from the rat S9, we noticed that this matrix strongly affects the recovery rates. To be sure that the products resulted from enzymatic reactions and were not due to any other process, we confirmed that no hydroxylated metabolites were detected with inactivated S9. Concerning the metabolites formed, we did not observe any species difference between rat and human, suggesting that similar Phase I enzymes are probably involved in the PTX-2 metabolism in mammals. Although PTX-2 metabolism has already been investigated, very little information has been published regarding the enzymes and transporters involved in PTX-2 uptake, metabolism, and excretion. Our results on gene expression revealed that PTX-2 could affect the regulation of several XME genes in human HepaRG cells. A pronounced up-regulation of CYP1A1 and 1A2 mRNA was indeed observed, indicating a plausible key role for these two enzymes in the hydroxylation of PTX-2. The upregulation of SULT1E1 and several UGTs suggests that PTX-2 itself or the hydroxylated metabolites formed through the CYP450 process may be conjugated. The results on transporter gene expression highlighted the possible role of P-gP and ABCG2 in the efflux of PTX-2, as these genes were found to be upregulated.

The induction of CYP1A mRNA could be correlated with the induction of protein levels, since we showed CYP1A2 induction using two different methodologies (immunostaining and western blot), and CYP1A1 induction via western blotting. However, we did not detect any increase of EROD activity in the HepaRG cells (up to 64 nM PTX-2 for 24 h or 48 h). We previously showed that five main CYP450 activities were not affected after 72 h of treatment with 5 nM PTX-2 [11]. It is possible that the level of increase of CYP1A mRNA had no impact on CYP activities. For instance, Genies et al. [23] showed that 200 nM of B[a]P treatment in HepG2 cells upregulated CYP1A1 mRNA at 6 h (200-fold induction), before decreasing drastically after 14 h of treatment. Despite strong mRNA induction, the EROD measurements at 24 h showed only weak CYP1A1 activity. Therefore, it can be assumed that the nM range of PTX-2 is too weak to cause an increase in CYP1A activities. Further investigations are needed to reveal the underlying mechanisms.

Using transactivation assays, we showed that PTX-2 activated neither CAR nor PXR. A previous study showed that PTX-2 failed to induce PXR translocation in HepG2 cells [11]. These results are in accordance with our data on gene expression, as CYP3A4, known to be primarily under PXR regulation [17], was not upregulated with PTX-2. For AhR, our model was biased by PTX-2 and we could not achieve a conclusion. Still, it is unlikely that PTX-2 would not interfere with AhR, as CYP1A1 and 1A2 gene regulation have been described as being regulated almost exclusively by AhR [14,15]. Another common methodology to study the possible activation of AhR by PTX-2 would be in silico modeling of the quantitative structure-activity relationship (QSAR) based, for instance, on protein X-ray crystallography. Although informative, this technique is a non-cellular model and needs to be confirmed in a cell-based assay.

Guo et al. [24] showed that hydroxylated metabolites of okadaic acid (OA) kept similar Protein Phosphatase 2A inhibition properties as the parent toxin. Besides, bioactivation has been previously reported for OA [25,26]. Using HepG2 transformed cell lines, Hashizume et al. [26] could also pinpoint the role of CYP1A2 in OA bioactivation. In light of this, the question arises as to whether the hydroxylated metabolite(s) of PTX-2 could produce the same effects as PTX-2 itself. In our study on metabolically competent HepaRG cells, PTX-2 toxicity was depicted with concomitant CYP1A2 induction. However, in cell models with no or low CYP1A levels (HEK-T and HepG2), no toxicity was observed, suggesting that the toxicity could be due to the formation of toxic Phase I metabolites. Further investigation is needed to confirm this hypothesis.

No previous study has been conducted to identify the CYP(s) responsible for PTX-2 metabolism. Ferron et al. [11] observed that the toxicity of PTX-2 on HepaRG cells was modified when CYP3A4 was chemically modulated by an inducer or an inhibitor. However, the authors questioned the specificity of the CYP3A4 inducer and inhibitor in such a complex cell model, and suggested that P-gP could also play a role in the toxic responses observed. From our results, we can suggest that PTX-2 is an inducer of its own metabolism, implying that CYP1A1 and CYP1A2 would be responsible for its hydroxylation. Such a phenomenon has already been described for several compounds [27,28]. The use of HepG2 transformed cell lines, as developed by Hashizume et al. [26], would be an appropriate way of confirming this assumption.

The elucidation of the structure of metabolites, as well as further investigation regarding the possible involvement of Phase II metabolism, is also needed to complete the available data on PTX-2.

## 4. Conclusions

In conclusion, we showed that PTX-2 undergoes Phase I metabolism with human S9 fractions, and at least one hydroxylated metabolite could be found. We also observed that PTX-2 up-regulates both CYP1A1 and 1A2 gene expression and induces CYP1A protein levels in HepaRG cells. No effects on several other CYP450s could be observed, which is consistent with the absence of CAR and PXR transactivation after PTX-2 treatment. To our knowledge, this is the first time that such a complete investigation of hepatic xenobiotic metabolism has been assessed for a phycotoxin.

## 5. Materials and Methods

### 5.1. Chemicals

PTX-2 standard was purchased from the National Research Council Institute for Marine Biosciences (Halifax, NS, Canada). Omeprazole, rifampicin, 3-methylcholanthrene, SR12813, CITCO, ethoxyresorufin, resorufin, and formate ammonium were purchased from Sigma-Aldrich (St. Louis, MO, USA). Reduced nicotinamide adeninedinucleotide phosphate (NADP+), glucose 6-phosphate (G6P), magnesium chloride hexahydrate, potassium chloride, Na_2_HPO_4_, and NaH_2_PO_4_ were purchased from Carl Roth (Karlsruhe, Germany). All of the other chemicals, including acetonitrile (ACN), methanol (MeOH), and dimethyl sulfoxide (DMSO) were of analytical grade and purchased from Fisher Scientific (Leicestershire, UK). Formic acid was purchased from Merck (Darmstadt, Germany). The deionised water was prepared using a Milli-Q system (Millipore, Bedford, MA, USA). The β-naphtoflavone and phenobarbital-induced Sprague Dawley rat and human hepatic S9 fractions were purchased from Biopredic International (Rennes, France).

### 5.2. S9 Phase I Metabolism

In order to target Phase I metabolism, specific co-factors were added to the S9 fractions: NADPH regenerating system (NADP+ (4 mM) and G6P (5 mM)), KCl (33 mM), MgCl_2_ (8 mM), and 0.1 M sodium phosphate buffer (Na_2_HPO_4_ [0.2 M] + NaH_2_PO_4_ [0.2 M], pH 7.4). An experimental volume of 0.5 mL containing Phase I co-factors (final concentration as described above), S9-fraction (final concentration 2.2 mg/mL), and 50 nM PTX-2 was incubated in a water bath at 37 °C for 3 h. The reaction was then stopped by adding 0.5 mL of ice-cold MeOH. After 20 min centrifugation (14,000 g) at 4 °C, the samples were analyzed or frozen at −80 °C until analysis. For the S9 controls, the same procedure was followed, but the S9 fraction was heat-inactivated for 45 min at 60 °C prior to incubation with the co-factors and PTX-2.

### 5.3. LC–HRMS Analysis

The metabolism investigation was conducted in two steps: first the decrease of the parent compound was measured via an LC–HRMS quantitative method, and then the formation of metabolites was studied via the metabolite research software MetWorks^®^. The analyses were conducted on the Thermo Fisher Accela LC (Thermo Fisher, Bremen, Germany) system hyphenated to an LTQ-Orbitrap XL mass spectrometer. The LC elutions were performed on an Agilent Zorbax Eclipse XDB-C18 column (Agilent Technologies, Santa Clara, CA, USA) (150 × 3.0 mm, 3.5 μm). Chromatographic separation was carried out using two mobile phase preparations, consisting of mobile phase (A), 100% water, and mobile phase (B), 5% water and 95% acetonitrile. Both mobile phases contained 2 mM of ammonium formate and 50 mM of formic acid. The gradient conditions were as follows: from 0 to 5 min, ramp up linearly from 98 to 2% of mobile phase A and hold for 7 min, then ramp back over 1 min to initial conditions and hold for 3 min to re-equilibrate the system. The flow rate was set at 0.3 mL min^−1^, the injection volume was 10 μL, and the column oven was maintained at 25 °C. PTX-2 was quantified using a calibration curve with PTX-2 standards at 0, 5, 10, 25, 50, 75, and 100 ng/mL in MeOH/H_2_O (2/3, 1/3). The mass spectrometer was operated with an electrospray ionization probe in positive mode using the following source parameters: sheath gas flow rate: 40 arb; auxiliary gas flow rate: 15 arb; sweep gas flow rate: 2 arb; ion spray voltage: 3.5 kV; capillary temperature: 350 °C; capillary voltage: 30 V; and tube lens: 100 V. The instrument was calibrated using the manufacturer’s calibration solution, consisting of three mass calibrators (i.e., caffeine, tetrapeptide MRFA, and Ultramark) to reach mass accuracies in the 1–3 ppm range. The instrument was operated in full-scan mode from *m*/*z* 100–1000 at a resolving power of 60,000 (full width at half maximum), allowing PTX-2 detection as ammonium adducts [PTX-2]-NH_4_+ (*m*/*z* = 876.51), as well as metabolite formation investigations using MetWorks 1.3.0. SP1. Software (Thermo Fisher Scientific, Waltham, MA, USA). The extraction’s mass window was set at ±5 ppm. The PTX-2 recoveries were calculated as follows: Ri = (ci × 100)/c0, where ci is the measured concentration of the sample i, and c0 is the initial concentration.

### 5.4. Cell Culture

#### 5.4.1. HepaRG Cells

HepaRG cells were cultured as previously published in [25,29]. Briefly, HepaRG cells (passages 13–19) were seeded at 30,000 cell/cm^2^ in 96-well plates in culture medium (Williams’ E Medium with GlutaMAX-I, supplemented with 10% fetal bovine serum, 100 IU/mL penicillin, 100 μg/mL streptomycin, 5 μg/mL bovine insulin, and 50 μM hydrocortisone hemisuccinate). After 2 weeks, the cells were cultured in the same medium supplemented with 1.7% DMSO (differentiation medium) for an additional 2 weeks. The medium was renewed every 2 to 3 days.

#### 5.4.2. HepG2 and HEK-T Cells

The human hepatocellular carcinoma cell line HepG2 and the human embryonic kidney cell line HEK-T were obtained from the European Collection of Cell Cultures (ECACC, Porton Down, UK). The cells were cultured in high glucose Dulbecco’s modified Eagle’s medium (DMEM, Pan-Biotech GmbH, Aidenbach, Germany), supplemented with 10% fetal calf serum (Pan-Biotech GmbH, Aidenbach, Germany), 100 U/mL penicillin, and 100 g/mL streptomycin (PAA Laboratories GmbH, Pasching, Austria) at 37 °C in a humidified atmosphere containing 5% CO_2_. The cells were passaged every 2–4 days (80–90% confluence), and seeded at 100,000 cells/cm^2^ and 50,000 cells/cm^2^ respectively for HepG2 and for HEK-T cells in 96-well plates.

### 5.5. Cytotoxicity Assays

Cell viability was assessed in HepaRG cells via the DAPI-mediated staining of nuclei. The cells were treated with different concentrations of PTX-2 for 24 h, and then fixed with 4% paraformaldehyde in Phosphate Buffered Saline (PBS) for 10 min and permeabilized with 0.2% Triton X-100. The nuclei were stained with 1 μg/mL DAPI, and quantified using ArrayScan (see below). Cell viability was determined in HepG2 and HEK-T cells after 24 h of treatment with PTX-2 using the CellTiter-Blue^®^ Cell Viability Assay (Promega, Madison, WI, USA). CellTiter-Blue^®^ reagent was diluted at 1:4 with phosphate buffered saline (PBS), and 20 μL of the diluted reagent was added to each well. The cells were incubated for 2 h at 37 °C, and the fluorescence was measured at 590 nm (excitation at 540 nm).

### 5.6. Real-Time Quantitative Polymerase Chain Reaction (RT-qPCR) Analysis

The HepaRG cells were seeded in 12-well plates at a density of 20,000 cells/cm^2^, and cultured until differentiation as described previously. Following 24 h incubation with PTX-2 or positive controls 50 μM omeprazole and 10 μM rifampicin, the cells were washed twice with Phosphate Buffered Saline (PBS). Total RNA extraction was then performed using the Total RNA isolation NucleoSpin^®^ RNA II kit from Macherey Nagel (Hoerd, France) following the manufacturer’s protocol. The RNA concentration and quality were determined by spectrophotometric measurements with BioSpec-nano (Shimadzu Biotech, Marne la Vallée, France). The RNA’s integrity was checked through electrophoresis using Experion (Bio-Rad, Marne la Coquette, France). The RNA samples were then reverse transcribed into double strand cDNA using the High Capacity RNA-to-cDNA kit (Applied Biosystems, Foster City, CA, USA) according to the manufacturer’s instructions. The sequences of target genes were obtained from the National Center for Biotechnology Information (NCBI) GenBank sequence database [30]. The primers were designed with the Primer designing tool from NCBI [31]. For each gene, at least one primer was designed on the exon–exon junction. All of the primers (see Table S2) were purchased from Sigma-Aldrich (Saint-Louis, MO, USA). Quantitative PCR was performed using a LightCycler^®^ 1536 from Roche (Mannheim, Germany). SYBR Green chemistry was used. The reactions were performed in a total volume of 2 μL containing 1X LightCycler 1536 DNA Green Master, 1× LightCycler 1536 DNA Master Mix (Roche), 300 nM of each primer, and 0.1 ng of cDNA. Negative quantitative PCR controls of RNase-free water were included in each run for a contamination assessment. The thermal cycling conditions were 94 °C for 15 s, followed by 40 cycles of 15 s at 94 °C, and 30 s at 60 °C with a slow temperature ramp (4.8 °C/s). LightCycler^®^ 1536 software (version 1.1.0.1112; Roche, Basel, Switzerland) was used for the quantitative analysis, and a melting curve analysis was used to check the specificity of each amplicon. The threshold Cqs were calculated from a baseline subtracted curve fit. Calibration curves were established for each gene from a serial dilution of a reference sample (pool of cDNA samples). According to these calibration curves, for each sample, mean relative amounts of mRNA of the target genes were calculated and then normalized to the GAPDH reference gene. The values were presented as fold changes relative to the solvent control.

### 5.7. CYP1A2 Protein Expression

After 24 h of incubation with PTX-2, the HepaRG cells were fixed with 4% paraformaldehyde in phosphate buffered saline (PBS) for 10 min and permeabilized with 0.2% Triton X-100 for 15 min. The plates were then incubated in blocking solution (PBS with 1% BSA and 0.05% Tween-20) for 30 min before the addition of primary antibodies prepared in blocking solution and filtered with a 0.2 μm syringe filter. The primary and secondary antibodies were purchased from Abcam (Cambridge, UK): mouse monoclonal anti-CYP1A2 S19 (ab22717) and goat anti-mouse IgG H&L DyLight^®^ (Thermo Fisher Scientific, Waltham, MA, USA) 550 (ab96876). The primary antibody (1/1000) was incubated overnight at 5 °C. After washing with PBS + 0.05% Tween 20, the secondary antibody (1/1000) was incubated for 45 min at room temperature. Nuclear DAPI (1 μg/mL) staining was used for automated cell identification by a high content analysis. The plates were scanned with the Thermo Scientific ArrayScan VTI HCS Reader (Thermo Scientific, Waltham, MA, USA), and analyzed using the Target Activation module of the BioApplication software (version: 6.0.1.4021; Thermo Fisher Scientific, Waltham, MA, USA). For each well, 10 fields (10× magnification) were scanned and analyzed for immunofluorescence quantification. Cell numbers were determined by cell counting following DAPI staining. CYP1A2 was quantified in the whole cell, and expressed as a fold increase compared to solvent control cells.

### 5.8. Western Blot for CYP1A Expression

Extracts from the HepaRG cells were separated using SDS-PAGE. The proteins were transferred to nitrocellulose membranes in a transfer buffer (25 mM Tris, 200 mM glycine, and ethanol 20%). The membranes were blocked in 5% low fat milk in Tris-buffer saline (TBS) (65 mM Tris pH 7.4, 150 mM NaCl) for 1 h at room temperature. The primary antibodies were purchased from Abcam (Cambridge, UK): mouse monoclonal anti-CYP1A2 S19 (ab22717) and rabbit polyclonal anti-CYP1A1 (ab3568), and from Santa Cruz Biotechnology (Dallas, TX, USA): mouse anti-HSC70 (SC-7298). The secondary antibodies were purchased from Dako (Santa Clara, CA, USA): goat anti-mouse (P0447) and goat anti-rabbit (P0448). The membranes were incubated with primary antibodies (1/1000) overnight at 4 °C. After being washed with TBS, appropriate secondary antibodies (1/1000) linked to horseradish peroxidase were incubated for one hour in 5% low-fat milk in TBS at room temperature. The immunocomplexes were visualized with an Immobilon Western Chemiluminescent HRP substrate (Millipore, Billerica, MA, USA), and scanned with a Fujifilm LAS-3000 imager (Fujifilm, Tokyo, Japan).

### 5.9. EROD Activity

The HepaRG cells were treated for 24 h or 48 h (renewal of PTX-2 after 24 h treatment) with PTX-2 in DMSO and a serum-free medium before the removal of the toxin and incubation with a specific CYP1A substrate. As a positive control, 3-MC (5 μM) was used. The CYP1A1/1A2 activity was monitored through a ethoxyresorufin-O-deethylase (EROD) reaction. The HepaRG cells were incubated with a solution of 2 μM ethoxy-resorufin for 30 min. The supernatants were then collected. The concentration of resorufin was determined by fluorescence measurement (λ ext = 530 nm and λ em = 585 nm). The protein content was measured with the bicinchoninic acid (BCA) assay (Pierce BCA Assay™ Kit, Thermo Fisher Scientific, Waltham, MA, USA). Activity is expressed as pmol of resorufin min^−1^ mg of protein^−1^.

### 5.10. CAR and PXR Transactivation Assays

The transactivation assays were conducted as previously described [32]. Briefly, 24 h after seeding, HepG2 cells were transiently transfected using TransIT-LT1 (Mirus Bio, Madison, WI, USA) according to the manufacturer’s protocol. For each well, the transfection mixture contained 40 ng pGAL4-(UAS)5-TK-luc, 40 ng pGAL4/DBD-hCAR/LBD(+3aa), and 1 ng pcDNA3-Rluc for the CAR assay. pcDNA3-Rluc was used as an internal control for normalization. Four to six hours after transfection, the cells were incubated with different concentrations of PTX-2 dissolved in serum-free DMEM without a phenol red medium. CAR agonist CITCO (10 μM) was used as positive control. After 24 h, the culture medium was removed and the cells were lysed after addition of 50 μL lysis buffer (100 mM potassium phosphate with 0.2% (*v*/*v*) Triton X-100, pH 7.8) for 15 min on an orbital shaker. After centrifugation (5 min, 2000 *g*) 5 μL of the supernatant was analyzed for luciferase activity as previously described [33]. The PXR transactivation assay was performed in HEK-T cells in the same way as that described for the CAR transactivation assay. The transfection mixture contained 40 ng pGAL4-(UAS)5-TK-luc, 40 ng pGAL4-hPXR-LBD, and 1 ng pcDNA3-Rluc per well. The PXR agonist SR12813 (10 μM) was used as a positive control. The firefly luciferase values were normalized to Renilla luciferase values, and expressed as fold-inductions normalized against solvent control.

### 5.11. AhR and CYP1A1 Reporter Gene Assays

The AhR and CYP1A1 reporter gene assays were performed in HepG2 cells as described for the CAR transactivation assay, except here no plasmid expressing a chimeric AhR was necessary, AhR basal expression being sufficiently high to measure reporter gene activity. For each well, the transfection mixture contained 80 ng p3xDREC for AhR or 50 ng pT81luc-hCYP1A1 for CYP1A1 and 1 ng pcDNA3-Rluc as an internal control for normalization. Four to six hours after transfection, the cells were incubated with different concentrations of PTX-2 dissolved in serum-free DMEM without a phenol red medium. The AhR agonist 3-Methylcholanthren (5 μM) and a mixture of 3-Methylcholanthren (5 μM) and CITCO (10 μM) for CYP1A1 were used as positive controls. The firefly luciferase values were normalized to Renilla luciferase values and expressed as fold-inductions normalized against solvent control.

### 5.12. Statistics/Data Analysis

GraphPad Prism 5 (GraphPad Software, Inc., La Jolla, CA, USA) was used for the statistical analyses. The data were compared to the control condition using one-way analysis of variance (ANOVA) followed by Dunnett’s post hoc tests. All error bars denote SD. Statistical significance was depicted as follows: * *p* < 0.05, ** *p* < 0.01, *** *p* < 0.001. 

## Figures and Tables

**Table 1:** Effects of PTX-2 on mRNA expression in HepaRG cells. The cells were treated with three sub-toxic doses of PTX-2 for 24 h. Rifampicin (RIF) (10 μM) and omeprazole (OME) (50 μM) were used as positive controls. The results were obtained from three independent experiments. The figures are the means ± standard deviations (SD) of fold change relative to solvent control. Fold change between 0.9 and 0.5 (light blue) or less than 0.5 (dark blue) depicts gene down-regulation whereas fold change between 1.0 to 2.5 (white), 2.6 to 8 (light red) or greater than 8 (dark red) depicts gene up-regulation. * *p* < 0.05, ** *p* < 0.01, *** *p* < 0.001 after one-way ANOVA followed by Dunnett’s post hoc tests.

Metabolism Phases	Gene	(nM)						OME		RIF		Gene
		16		32		64						
		Mean	SD	Mean	SD	Mean	SD	Mean	SD	Mean	SD	
**Nuclear receptors**	**AHR**	1.0	0.4	1.2	0.6	1.5	0.7	0.8	0.3	0.9	0.3	**AHR**
	**NR1I2**	1.3	0.2	1.5	0.6	1.4	0.3	1.0	/	0.7	0.4	**NR1I2**
**Phase 0 influx transporters**	**SLC22A1**	1.4	0.6	1.4	0.2	1.8	1.1	1.3	1.2	1.1	0.3	**SLC22A1**
	**SLC22A3**	1.6	0.3	1.8	0.4	2.4 **	0.3	1.0	0.4	1.1	0.5	**SLC22A3**
	**SLCO1A2**	0.9	0.6	1.2	1.1	1.1	0.6	0.4	0.3	0.2	0.2	**SLCO1A2**
	**SLCO1B1**	1.0	0.2	1.2	0.6	1.0	0.3	0.6	0.1	1.0	0.3	**SLCO1B1**
**Phase I mono-oxygenases**	**CYP1A1**	4.2	2.6	10.1	9.1	18.7	21.8	127.0 ***	67.1	0.4	0.4	**CYP1A1**
	**CYP1A2**	4.4	0.6	8.3	2.9	8.8	4.8	245.2 **	160.1	1.4	0.2	**CYP1A2**
	**CYP2B6**	1.6	0.5	3.1	2.1	3.5	2.6	8.0	8.0	4.3	2.5	**CYP2B6**
	**CYP2C9**	1.2	0.1	1.7	0.5	1.9	0.6	1.4	0.3	2.2 **	0.3	**CYP2C9**
	**CYP2C19**	1.3	0.3	1.4	0.2	1.8 *	0.3	1.2	0.2	1.6	0.4	**CYP2C19**
	**CYP3A4**	1.2	0.2	1.2	0.2	1.3	0.2	13.9 **	6.7	29.2 ***	3.6	**CYP3A4**
	**CYP3A5**	1.0	0.1	1.1	0.3	1.3	0.3	1.1	0.2	1.5*	0.2	**CYP3A5**
**Phase II transferases**	**GSTM1**	0.8	0.3	1.0	0.1	0.8	0.1	1.2	0.3	1.0	0.1	**GSTM1**
	**NAT1**	1.2	0.5	1.2	0.6	1.3	0.5	1.0	0.2	1.0	0.2	**NAT1**
	**NAT2**	1.1	0.0	1.2	0.1	1.3	0.3	0.8	0.2	0.9	0.3	**NAT2**
	**SULT1A1**	1.2	0.1	1.2	0.2	1.2	0.2	0.9	0.1	1.0	0.1	**SULT1A1**
	**SULT1E1**	1.7	1.2	2.4	2.0	3.5	3.4	0.4	0.2	0.7	0.4	**SULT1E1**
	**UGT1A1**	1.2	0.4	1.8	0.8	2.3	0.8	2.8	1.5	1.7	0.2	**UGT1A1**
	**UGT1A9**	1.3	0.3	2.1*	0.7	1.8	0.3	1.2	0.1	1.1	0.3	**UGT1A9**
	**UGT2B4**	1.6	0.6	2.1	0.7	1.7	0.5	1.3	0.2	1.4	0.5	**UGT2B4**
**Phase III efflux transporters**	**ABCB1**	1.4	0.2	1.9	0.6	2.1 *	0.6	1.4	0.5	1.7	0.4	**ABCB1**
	**ABCC2**	1.2	0.3	1.4	0.4	1.5	0.5	1.2	0.5	1.2	0.2	**ABCC2**
	**ABCC3**	1.0	0.1	1.3	0.3	1.4	0.3	1.0	0.3	0.9	0.0	**ABCC3**
	**ABCG2**	1.2	0.3	1.5	0.3	2.0	0.9	2.6	1.4	1.1	0.1	**ABCG2**
			0.4		0.9		2.5		8		250	
		x-Fold change compared to solvent control										

## Supplementary Materials

The following are available online at www.mdpi.com/2072-6651/9/7/212/s1. Figure S1: Cell viability in HepaRG cells. Following 24 h of treatment with different concentrations of PTX-2, the nuclei were stained with 1 μg/mL DAPI and scored using ArrayScan. The results were obtained from three independent experiments performed in triplicate (mean ± SD), Table S1: Effects of PTX-2 on mRNA expression of CYP1A1, 1A2, 2B6, and SULT1E1 in HepaRG cells. The data represent the means of fold change compared to solvent control, Table S2: Summary of primers used for q-PCR analysis.
