# Supplementary material for: Metabolism of the Marine Phycotoxin PTX-2 and Its Effects on Hepatic Xenobiotic Metabolism: Activation of Nuclear Receptors and Modulation of the Phase I Cytochrome P450
Source: Toxins (Basel). 2017 Jul 5;9(7):212. doi: 10.3390/toxins9070212 (PMC5535159; doi:10.3390/toxins9070212)
Supplement: Supplementary file 1 [file toxins-09-00212-s001.pdf]

Supplementary Materials: Metabolism of the Marine Phycotoxin PTX-2 and Its Effects on Hepatic Xenobiotic Metabolism: Activation of Nuclear Receptors and Modulation of the Phase I Cytochrome P450

Jimmy Alarcan, Estelle Dubreil, Antoine Huguet, Dominique Hurtaud-Pessel, Stefanie Hessel-Pras, Alfonso Lampen, Valérie Fessard, and Ludovic Le Hegarat

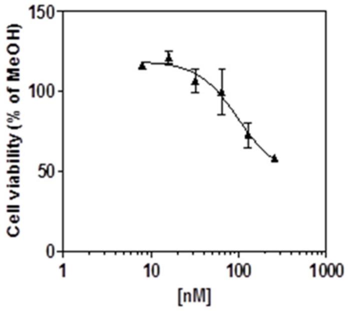

Figure S1. Cell viability in HepaRG cells. Following 24 h of treatment with different concentrations of PTX-2, the nuclei were stained with 1 µg/mL DAPI and scored using ArrayScan. The results were obtained from three independent experiments performed in triplicate (mean ± SD).

Table S1. Effects of PTX-2 on mRNA expression of CYP1A1, 1A2, 2B6, and SULT1E1 in HepaRG cells. The data represent the means of fold change compared to solvent control.

Gene	Assay	(nM)		
		16	32	64
CYP1A1	assay 1	2.7	4.7	5.5
	assay 2	2.8	4.9	6.7
	assay 3	7.2	20.6	43.8
CYP1A2	assay 1	4.7	5,0	8.2
	assay 2	3.7	9.1	13.9
	assay 3	4.8	10.7	4.3
CYP2B6	assay 1	1.1	1.9	1.9
	assay 2	2.2	5.6	6.4
	assay 3	1.4	2,0	2.1
SULT1E1	assay 1	1.3	1.7	7.4
	assay 2	0.8	0.8	1.1
	assay 3	3.1	4.6	2.2

Table S2. Summary of primers used for q-PCR analysis.

Gene	Sequence (5' to 3')		Gene	Sequence (5' to 3')	
AHR	F:	TAGGGTTTCAGCAGTCTGATGTC	SULT1E1	F:	ACAGGATCAACTAAACAGTGTACCA
	R:	CTACTGTCTGGGGGAGACCA		R:	ATCTGGTCTTGCCTGGAACG
NR1I2	F:	AGACACTGCAGGTGGCTTC	UGT1A1	F:	CTGCCTTCACCAAAATCCACTATC
	R:	TGGGGAGAAGAGGGAGATGG		R:	CACAGGACTGTCTGAGGGATTT
CYP1A1	F:	ACCCTGAAGGTGACAGTTCC	UGT1A9	F:	CGGAGTATGATCTCTACAGCCAC
	R:	TCTTGGAGGTGGCTGAGGTA		R:	TTCAAATTCCATAGGCAACGGC
CYP1A2	F:	CTTCGCTACCTGCCTAACCC	UGT2B4	F:	GAAGTTCTAGGAAGACCCACTACG
	R:	CCCGGACACTGTTCTTGTCA		R:	GGGTGAGGAAATTGAAAATCCCAG
CYP2B6	F:	TTCGGCGATTCTCTGTGACC	ABCB1	F:	CAGCTGTTGTCTTTGGTGCC
	R:	ATGAGGGCCCCCTTGGAT		R:	CCAATGTGTTCGGCATTAGGC
CYP2C9	F:	AAATGGAGAAGGAAAAGCACAACC	ABCC2	F:	GTGTGGATTCCCTTGGGCTT

	R:	TCAACTGCAGTGTTTTCCAAGC		R:	GAAGAAAACCAACGAATACCTGCTT
CYP2C19	F:	CCTGGAACGCATGGTGGT	ABCC3	F:	CCAACTCAGTCAAACGTGCG
	R:	TCCATTGCTGAAAACGATTCCAAAT		R:	ACCTAGGTTCTGCCAGAGGA
CYP3A4	F:	TCACAAACCGGAGGCCTTTT	ABCG2	F:	AGTTCTCAGCAGCTCTTCGG
	R:	TGGTGAAGGTTGGAGACAGC		R:	TTCCAACCTTGGAGTCTGCC
CYP3A5	F:	GCCCAATAAGGCACCACCTA	SLC22A1	F:	TGTCAAATTTGTTGGCGGGG
	R:	CCACCATTGACCCTTTGGGA		R:	TTTAACCAGTGCAGGTCAGGT
GSTM1	F:	GGGGGACGCTCCTGATTATG	SLC22A3	F:	GCATTGCTAAGTGCAATGGGA
	R:	GGGCAGATTGGGAAAGTCCA		R:	GCTTGTGAACCAAGCAAACATAAG
NAT1	F:	ACTAAGAAAGGGGATCATGGACATT	SLCO1A2	F:	GCACAAGAGTATTTGCTGGCAT
	R:	ACAGCTCGGATCTGGTGTTG		R:	CGGCAATCCGAGGTAGATGT
NAT2	F:	ACAGACCTTGGAAGCAAGAGG	SLCO1B1	F:	TCCACATCATTTTCAAGGGTCTACT
	R:	CTTCAATGTCCATGATCCCTTTGG		R:	TGCTTCATCCATGACACTTCCAT
SULT1A1	F:	TCGGAGAAGTGTCCTACGGAT	GAPDH	F:	GTCAAGGCTGAGAACGGGAA
	R:	CCACGAAGTCCACGGTCTC		R:	AAATGAGCCCCAGCCTTCTC
